# Employing Shadows for Multi-Person Tracking Based on a Single RGB-D Camera

**DOI:** 10.3390/s20041056

**Published:** 2020-02-15

**Authors:** Wei Gai, Meng Qi, Mingcong Ma, Lu Wang, Chenglei Yang, Juan Liu, Yulong Bian, Gerard de Melo, Shijun Liu, Xiangxu Meng

**Affiliations:** 1 School of Software, Shandong University, Jinan 250101, China; gaiwei1987@126.com (W.G.); chl_yang@sdu.edu.cn (C.Y.);; 2 School of Information Science and Engineering, Shandong Normal University, Jinan 250358, China; 3 Engineering Research Center of Digital Media Technology, MOE, Jinan 250101, China; 4 Department of Computer Science 110 Frelinghuysen Road Rutgers, The State University of New Jersey, Piscataway, NJ 08854-8019, USA

**Keywords:** multi-person tracking, RGB-D camera, shadow, occlusion

## Abstract

Although there are many algorithms to track people that are walking, existing methods mostly fail to cope with occluded bodies in the setting of multi-person tracking with one camera. In this paper, we propose a method to use people’s shadows as a clue to track them instead of treating shadows as mere noise. We introduce a novel method to track multiple people by fusing shadow data from the RGB image with skeleton data, both of which are captured by a single RGB Depth (RGB-D) camera. Skeletal tracking provides the positions of people that can be captured directly, while their shadows are used to track them when they are no longer visible. Our experiments confirm that this method can efficiently handle full occlusions. It thus has substantial value in resolving the occlusion problem in multi-person tracking, even with other kinds of cameras.

## 1. Introduction

In immersive virtual environments, locomotion through the virtual space is among the most crucial forms of interaction. The primary manifestation of human locomotion is walking, and, hence, genuine walking has substantial advantages over both virtual walking and flying as a mode of locomotion, in terms of its simplicity, straightforwardness, and naturalness [1]. Thus, it is not surprising that real walking in the physical space, which can engender greater degrees of flow experience and is preferred over non-moving modalities [2], has emerged as one of the most natural and effective interaction methods in virtual reality systems [3]. Moreover, it also increasingly serves as a natural means of interaction in many multi-player games. In the Interactive Tag Playground, for instance, the movement of players is tracked by four Kinect devices [4].

There are many algorithms seeking to track genuinely walking people, and visual tracking is a common method [5,6,7,8], of which Yilmaz et al. provide a good overview [9]. With regard to the methods used for visual tracking, RGB cameras are popular visual tracking devices relying on cues from color and intensity signals [10]. Recently, RGB Depth (RGB-D) cameras such as Microsoft’s Kinect, which is based on vision techniques, have enabled many new applications. They constitute a non-intrusive and appealing tracking technology due to their low cost and ease of deployment [6,11].

Unfortunately, one often faces the challenge of occlusion in multi-person tracking with a single front-view camera [6]. In recent years, many methods have sought to address this, including methods based on multiple cameras [12,13,14,15], Kinect setups relying on the ceiling [16], and approaches that fuse Kinect signals with other sensors [17,18]. However, these approaches may not be suitable in all settings, given issues such as their high cost or inconvenient deployment setup for users, and they do not solve the problem of occlusion during a long period of interaction or the problem of full body occlusion. Additionally, tracking-by-detection-based multi-person tracking is another commonly invoked approach, and the majority of such methods usually use an automatic pedestrian detector to obtain cropped pedestrian images. However, multi-person trackers are severely affected by misalignment in the pedestrian detectors, stemming from issues such as excessive background and missing parts, and occlusion. Heili et al. presented a conditional random field approach [19] for tracking-by-detection, exploiting long-term connectivity between pairs of detections and model similarities, as well as dissimilarities between them. In this method, the proposed confidence scores model the reliability of the features considering spatio-temporal reasoning such as occlusions between detections. Zhou et al. proposed an approach using a deep alignment network-based appearance model and a Kalman filter-based motion model to handle occlusion [20]. However, multi-person trackers cannot handle long-term occlusions.

In this paper, we assess to what extent shadows can serve as clues in tracking human movement. This is motivated by the fact that the shadow of a person moves in sync with a person’s body. Wang and Yagi also showed that shadows were helpful in pedestrian detection [21]. A person’s shadow exists in either indoor or outdoor conditions in many circumstances. In cases where such shadows are lacking, we can easily bring about shadows by adding a low-cost light source.

We propose a multi-person tracking algorithm fusing shadow signals in the RGB image with skeleton data, both of which are captured solely by a single RGB-D camera without any reliance on other sensors. Our experiments and sample application results show that our algorithm can resolve even long-duration and full-body occlusions using a single Kinect. This in turn helps to improve the tracking capability of the Kinect. The key contributions of our method are: (1) We propose a novel method to handle long-term occlusion by drawing on a person’s shadow. This is integrated into a tracking framework to compensate the reduction in the observation value, i.e., occlusion. (2) The method is a simple yet effective approach based on a single RGB-D camera without any other sensors. (3) We experimentally validate this approach.

The remainder of this paper is organized as follows: We begin by introducing related work. We then describe in detail our proposed algorithmic approach, followed by our experimental results, discussion, and conclusion.

## 2. Related Work

### 2.1. Multi-Person Tracking From RGB-D Data

Body occlusion is an important yet insufficiently well resolved problem in multi-person tracking. In this section, we introduce related work mainly with regard to tracking algorithms based on RGB-D cameras.

In recent years, the arrival of cheap RGB-Depth (RGB-D) devices such as Microsoft’s Kinect has facilitated the development of many new approaches to multiple person tracking. These sensors can provide color information as well as the estimated depth for each pixel [22]. RGB image and depth data are often used jointly as cues to resolve partial occlusion [23,24]. However, occlusion, especially full occlusion, is still a significant problem in real deployments of single, front-view camera systems.

Multiple cameras can be deployed to resolve full occlusions while tracking people [4,12,13,14,25,26,27]. With many cameras installed, when a person is occluded from view for one camera, said person may still remain visible via the other cameras, thereby lessening the chance of being occluded. “Out of Sight” is a toolkit that resolves the occlusion problem based on depth sensing [28]. The system can sense the tracking area from different angles by drawing on multiple Kinects. However, installing more cameras has a number of downsides, such as higher costs, difficulty in calibration, and an inconvenient deployment setup for users.

To address this problem, recent work has focused on the single perspective occlusion problem. An optimal camera placement scheme can aid in avoiding the full occlusion problem [16,29,30]. Wu et al. mounted a Kinect on the ceiling of a room so as to obtain a bird’s eye view to detect humans, wherein occlusions between people on the ground do not occur [16]. However, in settings with a high ceiling or without any ceiling, mounting a camera to obtain a bird’s eye view is either unfeasible or inconvenient.

Another approach to cope with occlusion under a single perspective is to rely on prediction methods based on motion trajectories, such as Kalman filters or particle filters [31,32]. Meshgi et al. proposed a tracker that exposes the particle filter to a probabilistic treatment of occlusions, fusing various features collected from the color and depth channels [33]. A binary flag (the occlusion flag) is tied to each particle in order to express the state of the tracker’s belief regarding the particle’s occlusion. However, long-duration occlusion from a single camera entails a loss of observation information, and thus these methods may fail to track the occluded person in the presence of long-duration or full occlusions.

Further research has proposed methods to fuse Kinect data with other sensor data [17,18,34]. Li et al. adopted a novel RFID-depth hybrid sensing approach to track both the identity and location of multiple people in groups [17]. As smartphones are ubiquitous, and most modern ones are equipped with an accelerometer and gyroscope, setups combining smartphones and other sensors have been considered [18,35,36]. Meng et al. presented a method that fuses smartphones and a depth-sensing camera to track people [18]. With the aid of a smartphone, when the user ends up out of the Kinect’s view, the system can detect that the user is still playing by analyzing the pattern of the smartphone’s sensor readings. None of the aforementioned methods fully address the long-duration and full occlusion problems adequately.

### 2.2. Shadow Detection

The person’s shadow has the same motion as the person. Therefore, we can treat the shadow as a separate moving object to detect and track [37]. The most widely adopted approach for moving object detection with fixed camera is background subtraction [38,39], and the moving object is detected based on the difference between the current frame and the current background model [40].

In recent years, deep learning has been widely used for shadow detection. Khan et al. first introduced deep Convolutional Neural Networks (CNNs) to automatically learn features for shadow regions and boundaries [41]. In the training phase, two CNNs are trained, one for labeling shadow regions and the other for labeling shadow boundaries. In the test phase, the predictions from both CNNs are combined into a unary potential for a Conditional Random Field (CRF) to label image pixels as shadow or non-shadow ones. Vicente et al. proposed a multikernel model for shadow region classification, and also embedded the multikernel region classifier into a CRF [42]. The parameters and hyperparameters of the model are efficiently optimized based on least-squares SVM leave-one-out estimates. However, these methods are mainly based on local region classifications. A recent approach for shadow detection introduces a Conditional Generative Adversarial Network (CGAN), wherein the generator of a CGAN has a full view of the entire image and can reason about the global structure and context [43]. Nguyen et al. presented the first application of adversarial training for shadow detection and developed a novel CGAN architecture with a tunable sensitivity parameter [44]. Wang et al. designed a framework based on a novel STacked CGAN (ST-CGAN) and presented a multi-task perspective. Compared with the existing work, it jointly learns both the detection and removal in an end-to-end manner such that the two objectives mutually benefit each other [45].

## 3. Multi-Person Tracking Method

In a front-view tracking system based on a single RGB-D camera, occlusion is a significant problem that can easily cause tracking to fail when tracking multiple people. Thus, it is necessary to design a powerful algorithm to avoid such tracking failures. In this section, we explore the basic idea and principle, and provide the details of our algorithm.

### 3.1. Basic Principle

As a popular RGB-D camera, Microsoft Kinect has one RGB camera, one infrared camera, and one infrared projector, to provide color, depth, and predicted skeleton data. The Kinect device with infrared camera and infrared projector modules is adopted for reliable human detection both in bright and in dark environments. The official Kinect software development kit (SDK) provides data in three spaces: the color image space, depth image space, and skeleton space. The depth image and skeleton spaces are in the camera coordinate system. The origin is the center of the infrared camera. The Z axis is the infrared camera’s axis and perpendicular to the image plane.

**Figure 1:**
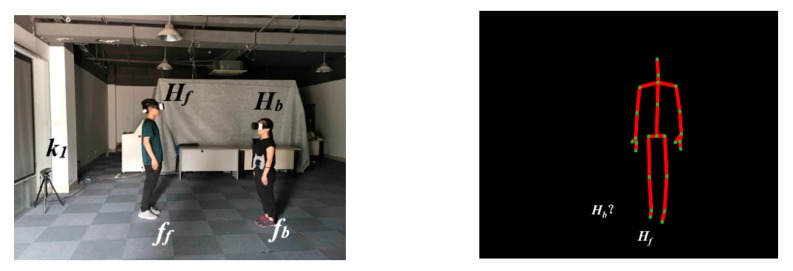
An example of an occlusion event.

In many applications, it has been shown that skeleton data can be reliably used to track people. Kinect V2 can predict the skeleton data of up to 6 people simultaneously. However, with a single Kinect, the skeleton of a person is lost when that person is occluded by others. Although RGB image and depth data of the Kinect can be used together as clues to resolve partial occlusions, these methods cannot handle complete body occlusions particularly well. In Figure 1, when the person *H_b_* is occluded by person *H_f_*, person *H_b_* cannot be seen from *k*_1_. In this case, the skeleton and joints of person *H_b_* cannot be detected for further tracking.

Fortunately, in such a case, shadows are cast that can easily be found. Such shadows are regions that are not directly reached by the light due to the obstruction by the human. They may exist in both indoor and outdoor settings. Therefore, shadows are informative in revealing the existence of the occluded person. Since the shadow of a person moves in conjunction with that person’s body, it can easily be captured from the RGB image of the Kinect, and thus it is possible to evaluate the position of the occluded person by analyzing their shadow. Furthermore, human shadows can also be expressly created by adding a light source, as a simple and low-cost solution.

**Figure 2:**
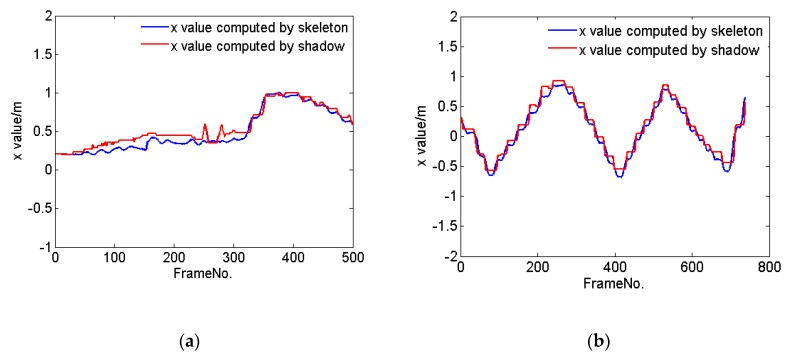
The trajectories of one person tracked by their skeleton (blue line) and shadow (red line), as the person walks along the X axis in the coordinate system of the Kinect (**a**) at various speeds, and (**b**) at a constant speed.

**Figure 3:**
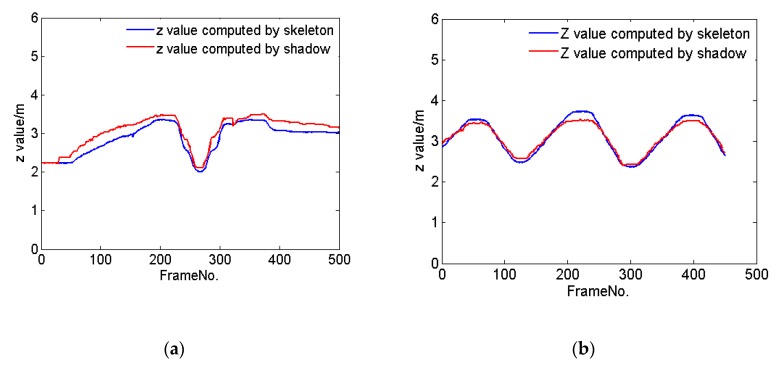
The tracking trajectories of one person computed using their skeleton (blue line) and shadow (red line), respectively, as the person walks along the Z direction in the coordinate system of the Kinect (**a**) at various speeds, and (**b**) at a constant speed.

**Figure 4:**
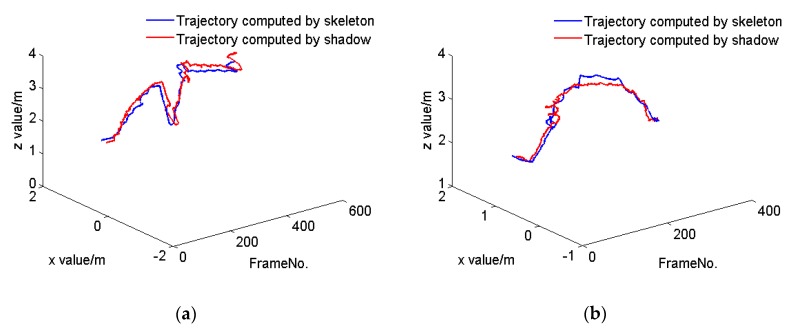
The tracking trajectories of one person computed using their skeleton (blue line) and shadow (red line), respectively, while they walk along arbitrary directions in the coordinate system of the Kinect (**a**) at various speeds, and (**b**) at a constant speed.

To investigate this, we tracked the center of mass (COM) of a person walking along the X axis in the coordinate system of the Kinect at a constant speed as well as at multiple speeds. Kinect V2 provides 25 joints of the human body, of which we selected the SpineBase joint to represent the position of the person. Figure 2 shows the tracking trajectories of the person computed by her skeleton (blue line) and shadow (red line). We further considered a person walking along the Z direction in the coordinate system of the Kinect at a constant speed and at various speeds. Figure 3 shows the resulting tracking trajectories for the person as computed by their skeleton and based on their shadow. Finally, Figure 4 shows the tracking trajectories of a person computed based on their skeleton and shadow, wherein the person walks at different speeds along different directions in the coordinate system of the Kinect. The results show that the position of the person computed by their shadow is close to that computed by their skeleton. Thus, we can rely on shadows as a valuable clue to assist in capturing a person’s position when their skeleton is lost, while continuing to rely on the skeleton data to compute the position of a person whenever such skeleton data is available.

Hence, the key idea of our algorithm is as follows: If there is no occlusion between people, the Kinect device can detect the person and provide skeleton data, and we can directly use it to track the person; otherwise, i.e., in the case that one person is occluded and cannot be detected by the Kinect, the skeleton data of the person is not available, so we make use of their shadow in the RGB image captured by the Kinect to assess the person’s position.

**Figure 5:**
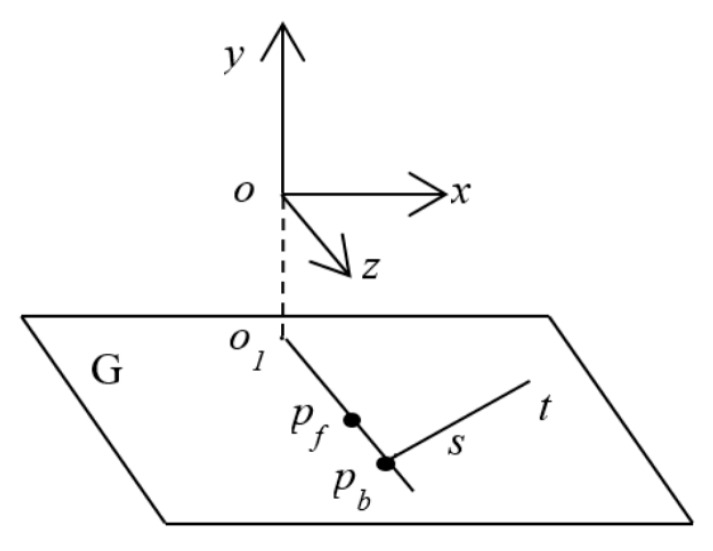
The skeleton space of the Kinect and the transformation relationship between RGB image space and skeleton space.

When relying on the shadow of a person to evaluate their location, it is necessary to segment the shadow within the RGB image and compute the position in the skeleton space. This requires a conversion between the image space and the skeleton space. In particular, Figure 1 shows an RGB image, in which *H_b_* is occluded by *H_f_*. Figure 5 shows the skeleton space, where *o* is the center of the Kinect infrared camera. *G* is the plane corresponding to the ground, parallel to the *xoz* plane. Here, *o*_1_ is the projection point of *o* on the ground plane *G*, and *p_f_* as well as *p_b_* represent the positions of the feet of *H_f_* and *H_b_*, respectively. Here, *p_f_*, *p_b_*, *o*_1_ are on *G*.

Since *H_b_* is occluded by *H_f_*, the points *o*_1,_ *p_f_*, *p_b_* are on the same line. In this case, *H_b_* is occluded by *H_f_*, but the shadow of *H_b_* is visible for the RGB camera. In Figure 5, the shadow of *H_b_* is represented by *st*. Therefore, we can compute the intersection point of *o*_1_*p_f_* and *st* on the plane *G*, which can be seen as *p_b_* to represent the position of *H_b_*.

**Figure 6:**
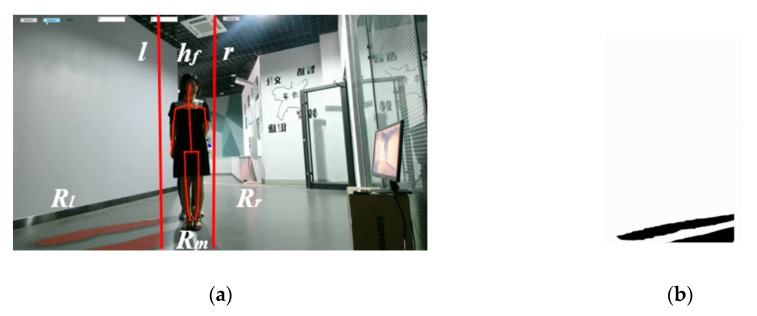
The region in which the occluded person’s shadows are located. (**a**) The RGB image is divided into three regions and (**b**) the difference image is obtained by subtracting the background image from *R_l_*. It includes the occluded person’s shadow.

Additionally, to quickly and efficiently extract the shadow of the occluded person, we need to locate the region in which the occluded person’s shadow can be found. In this paper, we assume that the light source and Kinect are placed such that the shadow of each person is on the left or alternatively on the right side of their body from the perspective of the Kinect during the tracking process. We let *a* = 0 or 1 designate the left or right side, respectively. For example, in Figure 1 and Figure 6, we can observe that the shadows of *H_f_* and *H_b_* are both located to their left.

As shown in Figure 6a, the lines *l* and *r* divide the RGB image into three parts *R_l_*, *R_m_*, and *R_r_*, where *R_l_* and *R_r_* are on the left and right side of *H_f_*, respectively. *R_r_* has no shadow, *R_l_* only has shadows of *H_f_* and *H_b_*, and the bodies and small parts of shadows of *H_f_* and *H_b_* are in the region *R_m_*. Hence, the region *R_l_* only has shadows of *H_f_* and *H_b_* after subtracting the background image (see Figure 6b). In this case, it is easier to extract shadows from *R_l_* than from the entire RGB image.

During the tracking process, if the shadow of *H_b_* appears in *R_l_* (when *a* = 0), then it will always exist in *R_l_*. Here, *R_l_* changes along with the changes of the position of *H_f_* in each frame (the method to compute these will be introduced in Section 4). Hence, we extract shadows from *R_l_*. Similarly, if the shadow of *H_b_* appears in *R_r_* (when *a* = 1), then it will always exist in *R_r_*. Here, *R_r_* changes along with any change in position of *H_f_* in each frame. Hence, we extract shadows from *R_r_*.

### 3.2. Algorithm Overview

In our method, when there is no loss of tracking, we detect the human body and obtain its skeletal model using the standard approach [46]. The skeleton is used to compute the person’s position. Otherwise, the shadow is used to track a person whose skeleton is lost in the tracking process.

In the initialization stage, the algorithm first captures the background image. For each person, once they are inside the depth-perceiving area of the Kinect, we begin to obtain their position using their skeleton data. Additionally, to quickly and efficiently extract the shadow of the occluded person when an occlusion occurs, we need to determine the region in which the person’s shadow is located, and set the value of a. During the main runtime stage, we attempt to obtain a person’s position using their skeleton data in each frame. If the skeleton of a person is found, we compute their position. Otherwise, we invoke our Shadow-based Tracking Algorithm (STA) to evaluate their position.

**Figure 7:**
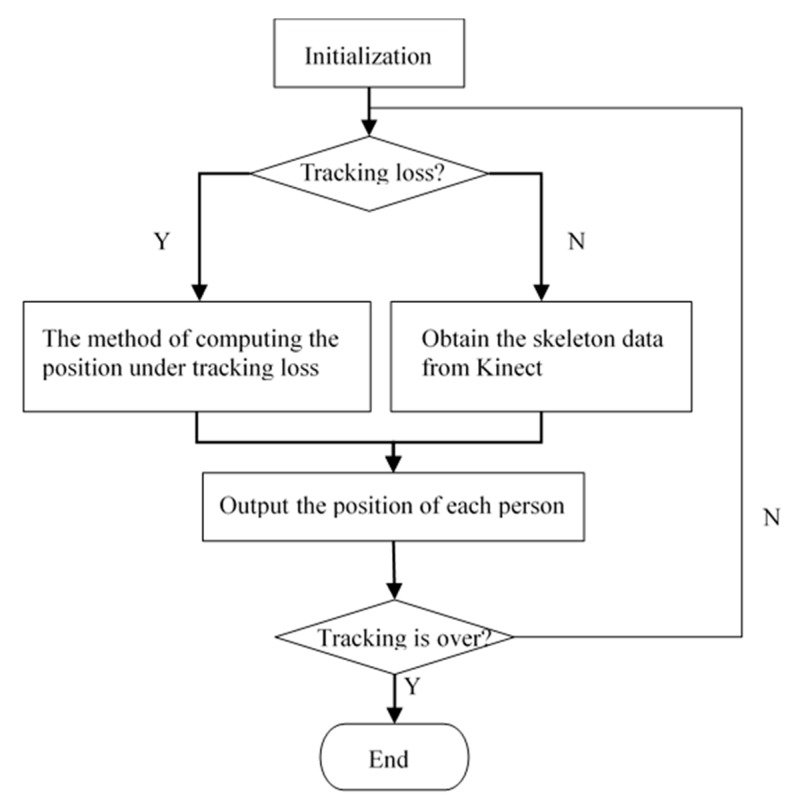
The flowchart of our proposed tracking method.

The details of our proposed tracking method are as follows (see Figure 7):

**Initialization**. Capture the background image as *B*, compute the number of people participating in the initialization scene, obtain each person’s position using the skeleton data obtained from the Kinect, and compute the value of *a* that indicates on which side of the person the shadow is located. 

**During the tracking, determine if tracking loss has occurred**. According to the difference of the number of users at adjacent time steps, determine whether any tracking loss has occurred. Assume the number of people at time *k* is *N_k_*. If *N_k+_*_1_ < *N_k_*, there is tracking loss, and we use the method of computing the position under tracking loss. Otherwise, we obtain the skeleton data of all target people from the Kinect.

**Compute the position under tracking loss**. There may be two cases in which a person has no skeleton: (1) The person is occluded by others. (2) The person is not occluded but walks out of the Kinect’s field of view. Based on the cause of tracking loss, we use the corresponding method to compute the position of the user. If the person is beyond the Kinect’s field of view, an audio feedback signal is played to warn them. At this moment, the user is requested to take steps backwards until the alarm clears, at which point we can get the position from the skeleton data. If, in contrast, the person is occluded by others, we use the proposed STA algorithm to evaluate the position.

**Output each person’s position**. Based on different methods, the position of each person can be computed. These are used as input for the rendering of virtual scenes later.

**Determine whether the tracking is over**. If so, finish the tracking; otherwise, continue to track users.

## 4. STA Algorithm Design and Implementation

For the case of a person being occluded by others, we invoke our novel STA Algorithm (Algorithm 1) exploiting shadows to solve the tracking under occlusion. In the following, we give an overview of our STA algorithm.
**Algorithm 1** Shadow-based Tracking Algorithm**Require:** A background image, current color image, and the skeleton data of *H_f_*. **Ensure:** The position of the occluded person *H_b_*. 1: Search the region *R* including the shadow in the current color image according to the value of   *a* that was computed in the initialization stage, and the skeleton data of *H_f_*; 2: Extract *H_b_*’s shadow in *R*; 3: Compute the position of *H_b_* based on the shadow; 4: **Return** position of *H_b_*

Assume that *H_b_* is occluded by *H_f_* (see Figure 1). First, we find the region encompassing the shadow of *H_b_* according to the value of *a* that we obtained in the initialization stage. Subsequently, we segment *H_b_*’s shadow. Thirdly, we compute a line representing the shadow and map the line onto the plane *G* in the skeleton space, designating it as *st*. Finally, we compute the intersection point *p_b_* between the lines *o*_1_*p_f_* and *st*, where *p_f_* and *p_b_* are the positions of *H_f_* and *H_b_*, respectively.

The specific details to implement this algorithm will be discussed next.

### 4.1. Search Shadow Region

We consider how to find the region *R* in accordance with the value of *a* computed in the initialization stage, which indicates which side of the person the shadow is on. We first obtain the head joint point of *H_f_* based on their skeleton data and transform it into the RGB image space using the function MapCameraPointsToColorSpace() provided by the Kinect SDK, marked as *h_f_* (see Figure 6a).

If *a* = 0, we consider the line *l*, which is a perpendicular line across the point (*h_f_.x* − *d_x_/*2, 0). The left region *R* of *l* is used to extract the shadow of *H_b_*. Otherwise, we consider the line *r*, which is a perpendicular line across the point (*h_f_.x* + *d_x_/*2, 0). The right region *R* of *r* will be used to extract the shadow of *H_b_*. Here, *d_x_* is evaluated according to the maximum width of the bodies of *H_f_* and *H_b_* in the initialization stage.

### 4.2. Extract Shadow

A pixel in the background subtraction results is considered as a possible shadow pixel when it has lower luminosity compared with the background image. We obtain the difference image C by subtracting the background image B from R:

For each pixel *R*(*x*, *y*) ∊ *R*, *C*(*x*, *y*) = *|R*(*x*, *y*) *− B*(*x*, *y*)|.

If *C*(*x*, *y*) *> T*, then *C*(*x*, *y*) is considered as belonging to the shadows of *H_f_* or *H_b_*, and we set *C*(*x*, *y*) = 1; otherwise, we set *C*(*x*, *y*) = 0.

In addition, each pixel can be presented using one Gaussian mixture model, and other methods such as texture or non-parametric representations may also be deployed [38,47].

### 4.3. Compute the Position of Occluded People via Shadows

Next, we consider how to compute the position of the occluded person. This entails computing the intersection point of *o*_1_*p_f_* and *st* on the plane *G* (see Figure 5).

**Figure 8:**
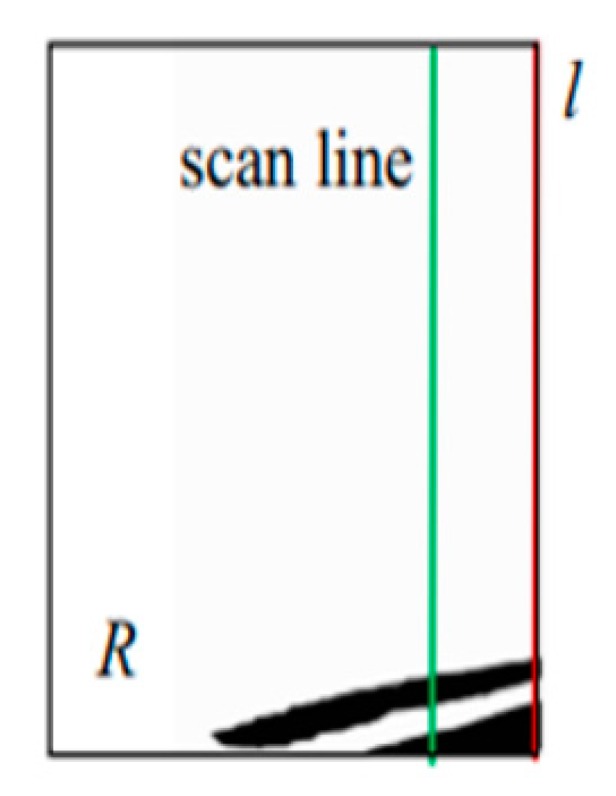
Determining the upper contour of *H_b_*.

**Figure 9:**
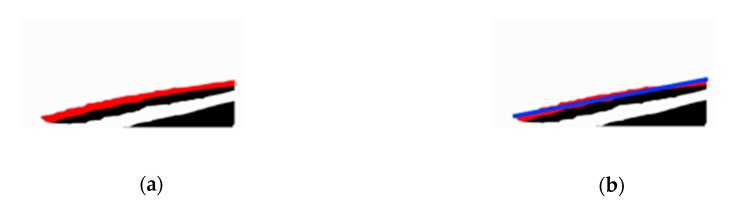
The result of fitting the upper contour of *H_b_* to a line: (**a**) the upper contour of *H_b_* (red line), (**b**) the fitted line (blue line).

First, we scan *R* from left to right. For each scan line, we access the pixel in *C* from top to bottom (see Figure 8). When we encounter the first pixels with a value of 1, we record this, stop the scan, and initiate the next scan. All such pixels together constitute the upper contour of *H_b_* (i.e., the red line in Figure 9a). Here, *S* is used to represent the set of points on the upper contour of *H_b_*. Then, we use the least squares method to fit this contour of *H_b_* to a line (the blue line in Figure 9b), and map it onto the plane *G*, which is *st*. Finally, we set the position of *H_b_* as the intersection point of *st* and *o*_1_*p_f_*. The method is described more formally in Algorithm 2, where W and H are the width and height of the RGB image, respectively. The origin (0,0) of coordinates is the top-left point in the RGB image.
**Algorithm 2.** Computing the Position of Occluded People via Shadows**Input:***a* represents which side of the user their shadow is located in, *h_f_* is the position of the head joint of *H_f_* in the RGB image, *R* is the search region, *C* is the binary image obtained by  background subtraction. **Output:** The position *p_b_* of *H_b_*. 1: S←∅ (stores points on the upper contour of *H_b_*) 2: **if**
*a* = 0 **then** 3:   *i*_1_ = 0, *i*_2_ = *h_f_*.*x − d_x_/*2 4: **else** 5:   *i*_1_ = *h_f_*.*x* + *d_x_*/2, *i*_2_ = *W* 6: **for** each *i* = *i*_1_ to *i*_2_
**do** 7:   **for** each *j* = *H* to 0 **do** 8:     **if**
*C*(*i*, *j*) = 1 **then** 9:       Add the point *R*(*i*, *j*) to *S*; 10:      Break; 11: Use the least squares method to fit the points in *S* to a line, and map it onto the plane *G*, designated as *st*; 12: Compute the intersection point of *st* and *o*_1_*p_f_*, marked as *p_b_*; 13: **Return** position *p_b_* of *H_b_*

## 5. Experimental Results

In order to verify the effectiveness and usability of our method, we conducted experiments using a Kinect sensor to track a set of human participants.

### 5.1. Experiment Design

In our experiments, we used two Kinect devices, one for tracking users and the other for evaluation purposes. We relied on Kinect *k*_1_ to track two human participants. In the tracking process, participant *H_f_* is always visible from *k*_1_, while for participant *H_b_*, the device may experience tracking loss. In order to evaluate the accuracy of our method, we used the secondary Kinect *k*_2_ to record the position of *H_b_*. Moreover, user *H_b_* was always visible from *k*_2_, and the trajectories obtained by *k*_2_ were used as reference values to test our method.

**Figure 10:**
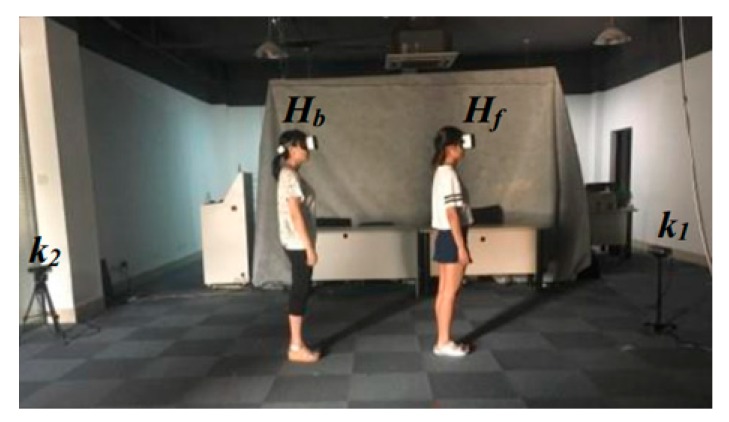
Example test environment.

The Kinects were placed at a height of 0.8 m above the ground, and the size of the tracking area was 5.7 m^2^. The relative positioning of the Kinect with respect to the human shadows is illustrated in Figure 10. We designed two experiments to assess the system. In the first experiment, we specifically evaluated the tracking accuracy when tracking is inhibited due to bodily occlusion. In the second experiment, we evaluated the tracking accuracy when the human participant moved freely, whereby the skeleton may have on occasion be tracked successfully, and on occasion may have failed to be tracked.

**Experiment 1.**  *The first experiment was designed to assess the accuracy of our method when person *H_b_* was occluded. In the experiment, the person was free to walk around, so that X and Z values could change. In order to better verify the accuracy of the algorithm, we considered three different runs along different paths. We first conducted runs leaving the position along one axis unchanged and allowing for the position along the other to change, so as to analyze the tracking accuracy with respect to an individual axis. In other words, the Z position changed, while the X position did not change, or vice versa. Subsequently, we allowed for changes along both axes while computing the position of the target. Accordingly, we designed three different motion paths depending on the direction of motion:*

*Path 1: When the points o*_1_ *p_f_ and p_b_ were approximately collinear, and the line o*_1_ *p_f_ was parallel to the Z axis, H_b_ moved back and forth along the Z direction, as in Figure 5. We analyzed the tracking accuracy with regard to the Z value when H_b_ was in full and long-duration occlusion.*

*Path 2: When a full-body occlusion occured, H_f_ and H_b_ moved back and forth along the X direction simultaneously. We analyzed the tracking accuracy of H_b_ with regards to the X value.*

*Path 3: When the points o*_1_, *p_f_ and p_b_ were approximately collinear and the line o*_1_*p_f_ was not parallel to the Z axis, H_b_ moved back and forth along the line o*_1_*p_f_. We analyzed the tracking accuracy of H_b_*.

We assessed each of these paths 10 times, relying on a pool of 5 human participants to assume the roles of *H_b_* and *H_f_*.

**Experiment 2.**  *In the second experiment, the participant H_b_ was instructed to move freely within the space. Compared with the first experiments, the user’s motion path had not been designed in advance. Thus, at any given instant, the skeleton of H_b_ may or may not be detected by the Kinect’s regular tracking algorithm. We verified the effectiveness of our method in various scenarios that may occur during person tracking, including non-occlusion and occlusion.*

### 5.2. Results

- Experiment 1

**Figure 11:**
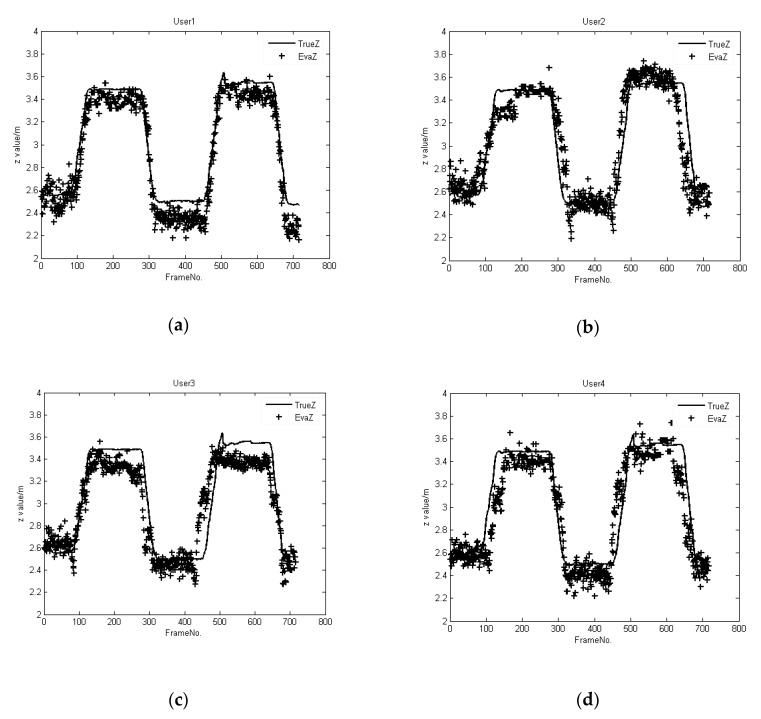
Comparison between trajectories as tracked by a person’s skeleton as opposed to computed using shadows for Path 1, where Users 1, 2, 3, and 4 are randomly selected human participants, and their tracking results correspond to (**a**–**d**), respectively.

There are three different motion paths in Experiment 1. The comparison between the position obtained via *H_b_*’s skeleton and the position computed via our shadow-based algorithm for Path 1 is given in Figure 11. Here, *H_b_* moves back and forth along the *Z* direction. The results show that there is only a minor deviation between the trajectories, as tracked by the participant’s skeleton and computed by our method, when the person is in long-term occlusion and full-body occlusion.

**Figure 12:**
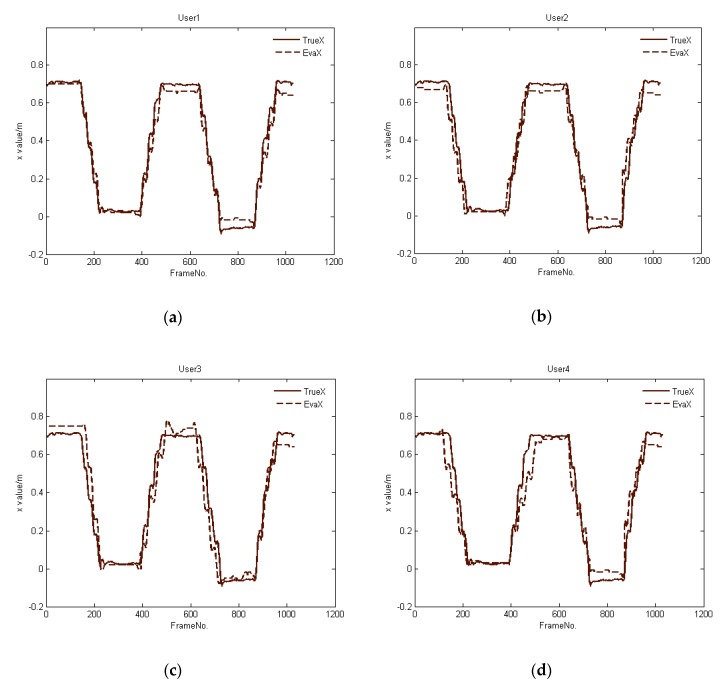
Comparison between trajectories as tracked by a person’s skeleton as opposed to computed using shadows for Path 2, where Users 1, 2, 3, and 4 are randomly selected human participants, and their tracking results correspond to (**a**–**d**), respectively.

Similarly, Figure 12 provides part of the tracking results for Path 2, recording *X* values of the occluded person *H_b_*. The results show that there is a minor deviation between the trajectories as tracked directly from *H_b_*’s skeleton, as opposed to those computed via shadows when the person is in full-body occlusion. When the person stops at a certain location, the tracking deviation of the algorithm is larger than while the person is in motion. Although there is a larger deviation when the person stops at a certain location, the deviation becomes smaller between the positions as tracked by the Kinect vs. as computed by our method when the user transitions from a stationary position to motion, which indicates that our algorithm has a small cumulative error.

**Figure 13:**
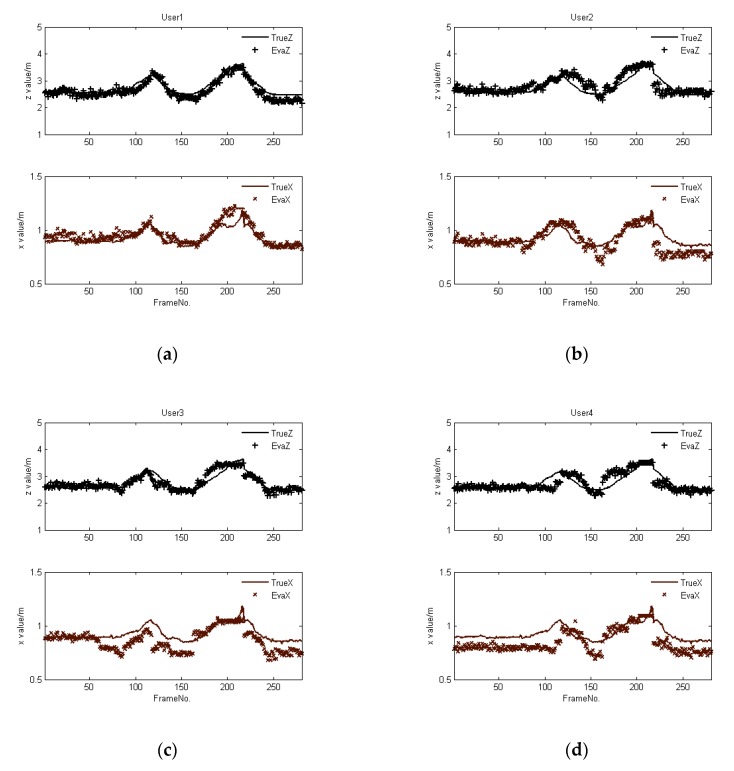
Comparison between trajectories as tracked by a person’s skeleton as opposed to computed using shadows for Path 3, where Users 1, 2, 3, and 4 are randomly selected human participants, and their tracking results correspond to (**a**–**d**), respectively.

For Path 3, the respective participant is free to walk around, entailing changes along both the *X* and *Z* axes. Figure 13 shows the obtained changes in both the *X* and *Z* directions. The results indicate that there is a minor deviation between the positions tracked by the person’s skeleton in comparison with those computed by our method.

Overall, the results suggest that our algorithm effectively computes the position of people, even when they are completely occluded or occluded for a long time, regardless of whether their position changes along a single axis or along both axes. Moreover, our algorithm is able to compute a person’s position effectively regardless of whether they are stationary, in motion, or in either of the two state transitions. This shows that our algorithm is robust in coping with a variety of occlusions.

- Experiment 2

**Figure 14:**
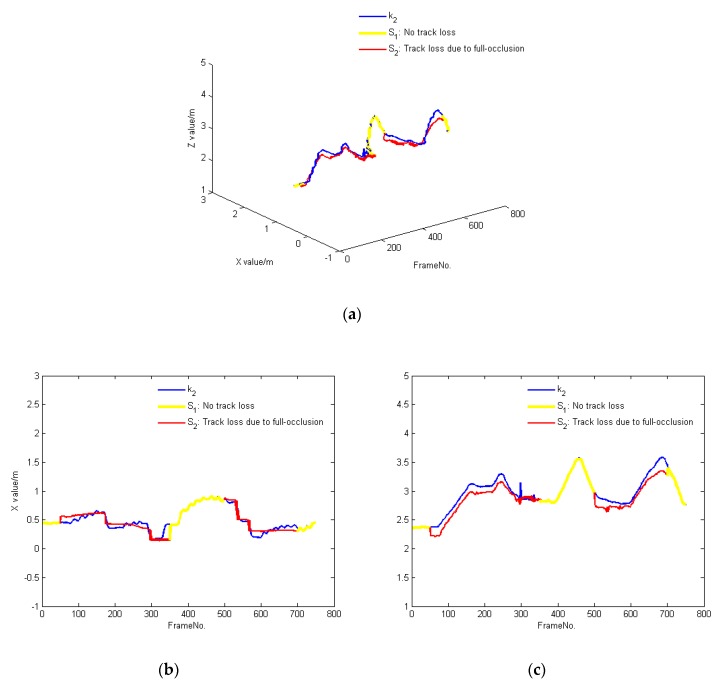
Comparison between trajectories as tracked by a person’s skeleton and via their shadow when *H_b_* moves in the tracking area, (**a**): The trajectories of a person B walking at random, (**b**): X value of the trajectories respectively at various speeds, (**c**): Z value of the trajectories respectively at various speeds.

We compared the tracking results obtained by the user’s skeleton data against the shadow-based tracking results of our algorithm when a person moves freely in the tracking area in Experiment 2. At any instance, *H_b_* may or may not be detected by Kinect *k*_1_, depending on whether there are occlusions. *H_b_*’s trajectory is recorded by a different Kinect *k*_2_, such that *H_b_* is always visible from *k*_2_. The trajectory of *H_b_* is represented by blue lines in Figure 14.

When there is no track loss, *H_b_* is detected by *k*_1_ in *S*_1_, so the obtained position is consistent with the position obtained by *k*_2_ in Figure 14. When there is track loss, the trajectory of *H_b_* is computed by our algorithm in *S*_2_, which is close to the position obtained by *k*_2_. Overall, when a person is in different tracking states, the trajectory obtained by our algorithm is very close to the actual trajectory of that person, which shows the effectiveness of our algorithm.

In Experiment 2, the participant can move freely, which means that various different situations may occur during tracking. To better assess the accuracy of our algorithm, we more explicitly measured the tracking accuracy in Experiment 2 to more quantitatively assess it.

In particular, we computed the deviation between the result of our method and the trajectory of the occluded person *H_b_* obtained from *k*_2_ as follows:

$$Err_{i}^{t}=\frac{\sum_{t=1}^{N_{F}}{\mathrm{err}}_{i}^{t}}{N_{F}}$$

$${\mathrm{err}}_{i}^{t}=\sqrt{{\left(p_{i}^{t}-q_{i}^{t}\right)}^{2}}$$

Here, $Err_{i}^{t}$ refers to the error value of the trajectory of *H_b_* at time *t*, *N_F_* refers to the duration of the entire tracking run, $p_{i}^{t}$ and $q_{i}^{t}$ respectively refer to the trajectory of *H_b_* at time *t* computed by our method and captured by Kinect *k*_2_.

Based on this, in order to evaluate the effectiveness of our method, we compute the accuracy as

$$acc_{i}=e^{-Err_{i}^{t}}$$

Hence, one obtains accuracy values in the range [0, 1], such that the smaller the error value, the higher the accuracy.

**Figure 15:**
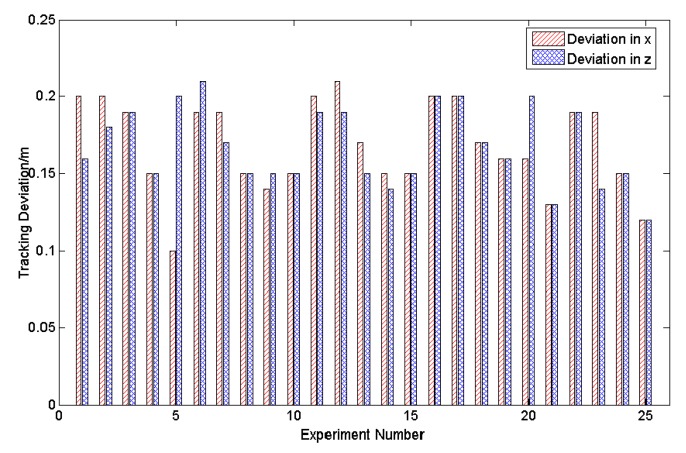
The tracking deviation between trajectories as tracked by a person’s skeleton vs. as computed by our method along the X and Z axes in Experiment 2, where 25 results selected randomly from 50 results are given.

First, we computed the tracking deviation of participants along the *X* and *Z* axes. The maximum tracking deviation along the X axis is 0.21 and the minimum tracking deviation is 0.14, while the average is 0.17. Similarly, the maximum and minimum deviations along the Z axis are 0.21 and 0.1, respectively, and the average is 0.16. Parts of the tracking results are shown in Figure 15.

**Figure 16:**
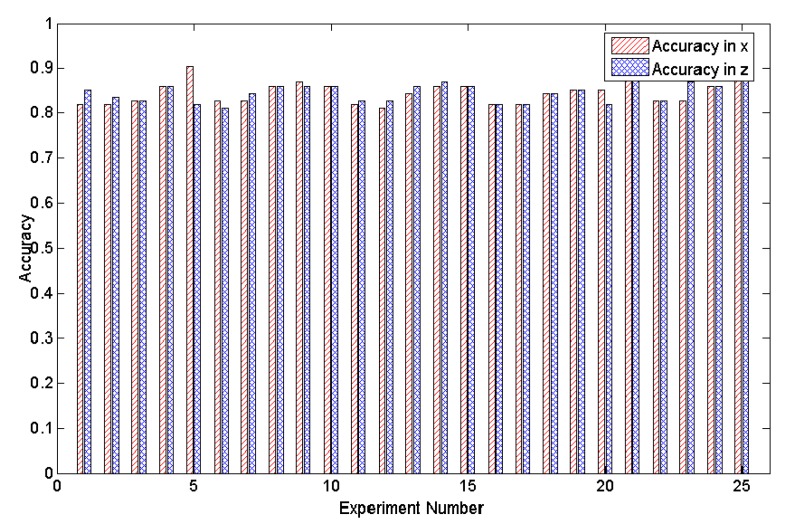
The tracking accuracy between trajectories as tracked by a person’s skeleton vs. as computed by our method along the X and Z axes in Experiment 2, where 25 results selected randomly from 50 results are given.

Subsequently, we measured the tracking accuracy based on the deviation (see Figure 16). The mean value of the tracking accuracy is 0.8 based on our method, which demonstrates that shadows can indeed be used to track the positions of people when their skeletons are lost.

**Figure 17:**
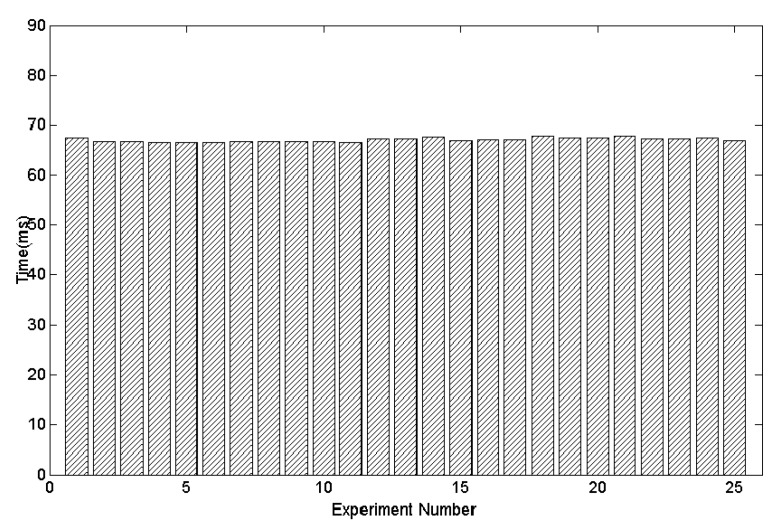
The time cost of 25 times by 5 users for an experimental task.

Additionally, we computed the computation time for our method. The algorithm is evaluated on a 2.8GHz Intel Core i5 computer, and parts of the tracking results are shown in Figure 17. The average time cost is 67 min for each frame, which is equivalent to about 15 frames per second (fps). This indicates that our proposed method is a feasible choice for real-time applications on modest hardware.

## 6. Discussion

Occlusion has been a persistent problem for multi-person tracking with a single view camera. Although a variety of tracking algorithms have been proposed, they do not effectively and efficiently solve the challenges presented by long duration and full-body occlusion. In this paper, we explore the idea of relying on shadows as additional cues in tracking body movement, rather than merely treating such shadows as noise. We found that shadows are informative in revealing the whereabouts of an occluded person. Wang and Yagi found that shadows are helpful in pedestrian detection [21]. Our findings are consistent with the previous study.

Based on these considerations, the problem of computing the motion of the occluded person is transformed into that of computing the shadow movement of the occluded person. Nevertheless, only little prior work has evaluated this issue in multi-person tracking.

**Figure 18:**
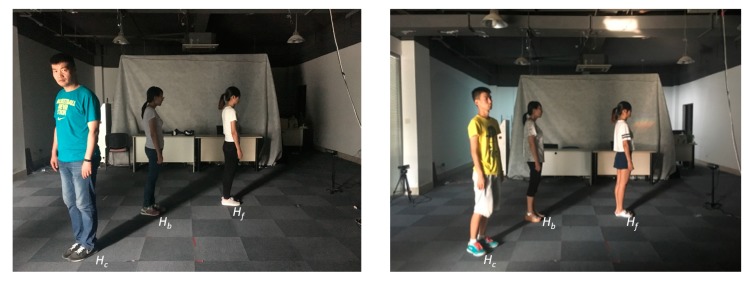
A special case of the occluded person *H_b_*.

In our proposed method, we focused on how to compensate for the reduction in the observable data. To this end, our method leveraged the user’s shadow as a feature to locate occluded subjects. Theoretically, it is also possible that *H_b_* was occluded by *H_f_* and that their shadow was covered by that of *H_c_* (see Figure 18). In this case, our method could still use the overlapping shadows to compute the position of *H_b_*. We computed the tracking accuracy and determined the mean value of the tracking accuracy as 0.8 based on our method.

In terms of limitations, the success of this method hinges on an accurate shadow detection, which implies that if the shadow is overly light, it will likely not be captured accurately. Our method is robust in the indoor setting, considering that this problem can be addressed by adjusting the lighting so as to obtain darker shadows. The method cannot deal with occlusion when there is no sunlight in an outdoor setting. To further improve the detection, we intend to integrate region of interests (ROI) segmentation [48] and Frustum PointNets [49] to design a more accurate multi-user tracking algorithm for a single RGB-D camera setup. Considering the fact that shadows tend to vary according to the relative position between a person and the light source, the ROI segmentation network will be used to learn to segment partial shadows given in the bounding box, which is different from conventional foreground segmentation networks that focus on segmenting the entire object.

Additionally, our experiments confirm that the proposed method can resolve long-duration and full-body occlusion between two people with a single Kinect. Our approach could also be extended to support multiple people. Furthermore, we only considered the case of a single shadow of a person for a given light source. In settings involving more than one shadow of a person, our method would need to adopt a more elaborate shadow tracking mechanism. Hence, the present study constitutes an initial exploration and opens up new avenues in exploring the potential of shadows for tracking purposes.

## 7. Conclusions

In this paper, we proposed a method fusing shadow and skeletal data to track two people using just a single Kinect device. Our experiments show that our algorithm can cope with both long-duration occlusion and full-body occlusion. Our experiments demonstrate that one can improve the tracking capabilities for people in motion with a single Kinect, without needing to resort to the use of additional sensor devices. 

The system has recently been applied to mobile VR systems such as maze games as well as a firefighter training simulation system for two players in an indoor setting. In such types of VR applications, users wear a head-mounted display and move inside a specific area to explore the scenes and interact with the virtual world. Furthermore, the method could be invoked as a complementary means of tracking people using other RGB-D cameras and video cameras.
